# Real-world evidence on first-line treatment for metastatic renal cell carcinoma with non-clear cell and sarcomatoid histologies: are sunitinib and pazopanib interchangeable?

**DOI:** 10.3332/ecancer.2019.973

**Published:** 2019-11-04

**Authors:** Renata Colombo Bonadioa, Pedro Isaacsson Velho, Guilherme Nader Marta, Mirella Nardo, Manoel Carlos LA Souza, David QB Muniz, Regis OF Bezerra, Raisa KA Bispo, Sheila F Faraj, Diogo A Bastos, Carlos Dzik

**Affiliations:** Instituto do Cancer do Estado de São Paulo, Faculdade de Medicina da Universidade de São Paulo, Av Dr Arnaldo 251, São Paulo 01246-000, Brazil; a https://orcid.org/0000-0001-5818-922X

**Keywords:** non-clear cell, sarcomatoid, renal cell carcinoma, sunitinib, pazopanib

## Abstract

**Introduction:**

Non-clear cell renal cell carcinoma (nccRCC) and sarcomatoid renal cell carcinoma (sRCC) are underrepresented in clinical trials. Treatment approaches are frequently extrapolated from data of clear cell renal cell carcinoma, in which pazopanib is non-inferior to sunitinib. We aim to compare the effectiveness of first-line sunitinib and pazopanib for nccRCC and sRCC.

**Methods:**

We evaluated a retrospective cohort of patients with metastatic nccRCC and sRCC treated with first-line sunitinib or pazopanib at an academic cancer centre. Overall survival (OS), progression-free survival (PFS) and response rate were measured. Kaplan–Meier and log-rank analyses were used for time-to-event data. Cox regression was used for prognostic factors.

**Results:**

Fifty-three patients were included; 16 (30.1%) treated with sunitinib and 37 (69.9%) with pazopanib. Forty-six (86.8%) patients had nccRCC and 7 (13.2%) had sRCC. The majority had intermediate or poor International Metastatic Renal-Cell Carcinoma Database Consortium risk (93%).

Median PFS was 6.6 months with sunitinib and 4.9 months with pazopanib (HR 1.75; *P* = 0.078). Treatment with pazopanib was associated with inferior OS in comparison with sunitinib (median OS: 30.4 months versus 8.7 months; HR 2.71, 95% CI 1.31–5.58, *P* = 0.007). These results were confirmed in subgroup analysis of patients with papillary, chromophobe and MiT family translocation histologies (median OS: 38.7 months versus 14.7 months; HR 3.16, 95% CI 1.20–8.29, *P* = 0.019). Unclassified and sarcomatoid histologies had inferior OS (median: 6.9 and 1.1 months, respectively) regardless of the treatment used.

**Conclusion:**

In this patient cohort, pazopanib was associated with inferior OS in comparison with sunitinib for metastatic nccRCC. Larger trials are ideally warranted to confirm these results.

## Introduction

Renal cell carcinomas (RCCs) represent more than 80% of all primary renal neoplasms [1]. Clear cell RCC (ccRCC) comprises 85% of the RCC. Other RCC variants are classified as non-clear cell RCC (nccRCC), a heterogeneous group of neoplasms composed of several histological variants [2]. Although they are commonly grouped together, their morphological and molecular differences significantly impact clinical outcomes and prognosis [3, 4]. Sarcomatoid RCC (sRCC) also represents a small portion of RCC, having an aggressive phenotype and may be present in each different histologic epithelial subtypes [5].

Despite several advances that have been achieved in recent years in the treatment of metastatic RCC, clinical data derives predominantly from patients with ccRCC [6]. Most of the pivotal studies leading to the approval of the current standard therapies for RCC included a limited number of or completely excluded nccRCC and sRCC patients [7, 8]. The scarcity of clinical evidence regarding nccRCC and sRCC limits our knowledge on the efficacy and safety of therapies in these different variants. Thus, many management recommendations are extrapolated from existing evidence for ccRCC.

For patients with metastatic nccRCC, the standard first-line treatment options include angiogenesis inhibition with vascular endothelial growth factor (VEGF) pathway inhibitors and mammalian target of rapamycin (mTOR) inhibitors [9, 10]. Temsirolimus is an mTOR inhibitor that was compared with interferon-alpha for advanced renal-cell carcinoma in a phase III trial, which included patients with nccRCC. Results showed superior progression-free survival (PFS) and overall survival (OS) with temsirolimus, regardless of tumour histology [11, 12].

Additional evidence is available from phase II trials, such as the ASPEN trial, which randomly assigned patients with nccRCC to receive either sunitinib or everolimus in the first-line setting. Median PFS was longer in patients randomised to sunitinib (8.3 months versus 5.6 months, HR 1.41, 80% CI 1.03–1.92, *P* = 0.16) [13]. A second trial, the Everolimus versus Sunitinib Prospective Evaluation (ESPN), randomly assigned patients with nccRCC to treatment with everolimus or sunitinib, with crossover to the alternate agent at disease progression. No statistically significant differences in survival were seen between the two treatment arms (median OS: 16.2 months versus 14.9 months, 95% CI 8.0–23.4, stratified *P* = 0.18) [14]. Finally, the phase II RECORD-3 study evaluated the treatment sequencing with everolimus followed by sunitinib versus sunitinib followed by everolimus. A subset analysis of nccRCC patients failed to demonstrate a statistically significant difference in PFS between the two arms (median PFS: 7.2 months versus 5.1 months, HR 1.5, 95% CI 0.9–2.8) [15].

Results of a recent meta-analysis of randomised controlled trials have suggested a trend towards favouring sunitinib in terms of OS and PFS [16]. However, the results were not statistically significant. Thus, optimal therapy for nccRCC histologies remains unclear. Pazopanib is an anti-VEGF tyrosine kinase inhibitor (TKI) frequently used for RCC. Although pazopanib is non-inferior to sunitinib for ccRCC, evidence on its use for nccRCC and sRCC is scarce [17]. We sought to analyse our centre’s experience regarding the effectiveness of sunitinib or pazopanib as first-line therapy for metastatic nccRCC and sRCC.

## Methods

### Study design and participants

We retrospectively evaluated data from 222 consecutive patients who received sunitinib or pazopanib as initial systemic treatment for metastatic RCC from February 2009 to March 2017 at Instituto do Cancer do Estado de São Paulo (ICESP), Brazil. The use of sunitinib or pazopanib depended only on the time period. From February 2009 to September 2013, sunitinib was the TKI inhibitor available as first-line therapy for metastatic RCC at ICESP. From September 2013 until March 2017, pazopanib was the TKI inhibitor available.

Only patients with histological confirmation of nccRCC who received at least one dose of the VEGF inhibitor were included in the present analysis. One specialised pathologist centrally reviewed the tumour samples available to confirm the histopathological diagnosis.

Clinical records were evaluated in order to collect relevant data including age, gender, histology, Karnofsky performance status, prior nephrectomy, sites of metastatic disease and prognostic score according to the International Metastatic Renal-Cell Carcinoma Database Consortium (IMDC) system [18].

Two trained radiologists reviewed all radiologic imaging (computed tomography or magnetic resonance imaging) to assess tumour response, according to RECIST 1.1 [19].

### Treatment

Pazopanib was administered orally at the dose of 800 mg or lower once daily (600 or 400 mg once daily). Sunitinib was administered orally at the dose of 50 mg or lower once daily, for treatment cycles of 4 weeks on treatment and 2 weeks off treatment, or at 37.5 mg once daily continuously. Treatment continued until disease progression, unacceptable toxicity, patient refusal or death.

### Statistical analysis

The primary objective of the study was to compare the OS of patients who received pazopanib versus sunitinib. Secondary objectives were response rate, PFS and prognostic factors.

OS was defined as the time from initiation of systemic treatment to death. PFS was the time from treatment initiation until clinical or radiological progression, or death from any cause. Patients without the described events were censured at the time of the last follow-up.

Continuous variables were expressed as median and ranges, and compared using the unpaired *t*-test. Categorical variables were expressed as absolute and relative frequencies, and compared using the Chi-squared test or Fisher’s exact test. The response rate during treatment duration (complete response, partial response, stable disease or progressive disease) was compared between patients receiving sunitinib versus pazopanib with Fisher’s exact test.

Survival curves were estimated with the Kaplan–Meier method. Analyses of differences between survival curves were performed using the log-rank test. Prognostic factors were evaluated with univariable and multivariable analyses using the Cox proportional hazards model. Variables were included in the multivariable analyses if they presented a *P* value < 0.10 in the univariable analysis and were not associated with each other.

Statistical significance was defined by *P* values < 0.05. Statistical analyses were performed with Stata software, version 14 (StataCorp, Texas, USA).

## Results

### Patients’ characteristics

A total of 53 patients with metastatic nccRCC that received first-line sunitinib or pazopanib were identified. Sixteen (30.1%) patients received first-line treatment with sunitinib and 37 (69.9%), with pazopanib.

**Figure 1.**
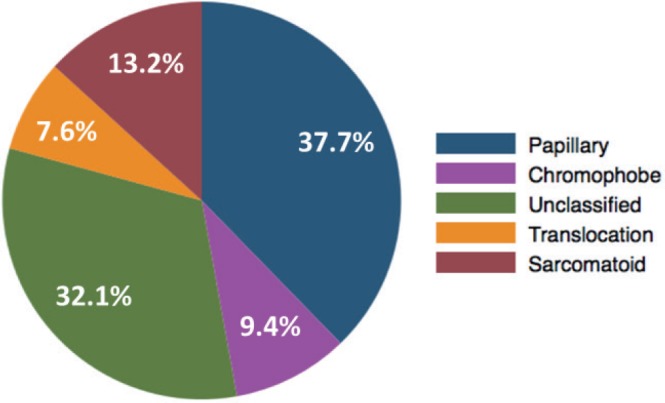
Pie chart of the distribution of histologic subtypes of nccRCC and sRCC in the study population.

Median age was 56.3 years old (range 16.9–79.1). The predominant histologic subtypes were papillary (20 patients, 37.7%) and unclassified (17 patients, 32.1%). Other histologies that were present in smaller numbers were sarcomatoid differentiation (7 patients, 13.2%), chromophobe (5 patients, 9.4%) and MiT family translocation (4 patients, 7.6%). Central pathology review by a specialised urological pathologist resulted in the reclassification of two cases: one was reclassified as clear cell carcinoma (and, therefore, excluded from the present study), and another was reclassified from papillary to MiT family translocation nccRCC. The final histologic subtypes distribution is the one described here and presented in Figure 1.

All demographic, clinical and pathological characteristics of our patients are detailed in Table 1. The two treatment groups differed in terms of histologic subtypes, with a higher proportion of patients with unclassified and MiT family translocation histologies in the pazopanib group (*P* = 0.023). Other clinical and demographic characteristics were balanced between the two groups.

### Treatment efficacy

Thirty-five patients had adequate imaging available prior and after TKI treatment for evaluation of response according to RECIST 1.1. The response rate did not differ between the two groups (*P* = 1.000). Partial response occurred in 8.3% (*N* = 1) with sunitinib and 8.7% (*N* = 2) with pazopanib, while stable disease occurred in 66.6% (*N* = 8) and 60.8% (*N* = 14) and progressive disease in 25% (*N* = 3) and 30.4% (*N* = 7), respectively.

**Figure 2.**
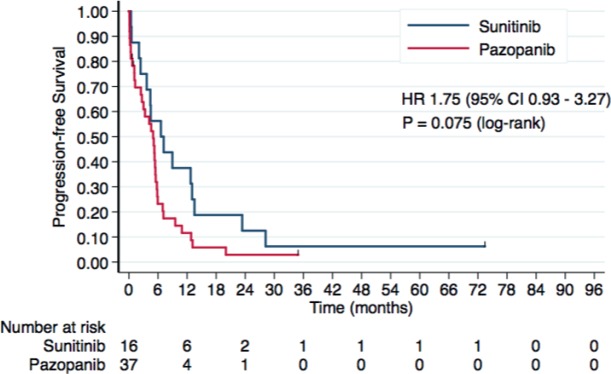
Kaplan–Meier curves for PFS according to the treatment group in the overall study population.

Forty-nine patients presented disease progression or death. Median PFS was 6.6 months with sunitinib and 4.9 months with pazopanib (HR 1.75, 95% CI 0.93–3.27, *P* = 0.078). Kaplan–Meier curves for PFS are presented in Figure 2.

**Figure 3.**
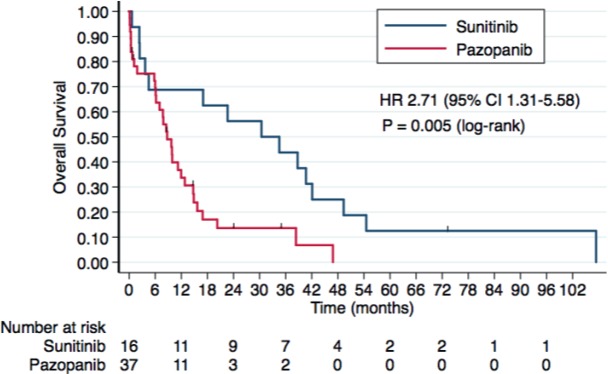
Kaplan–Meier curves for OS according to the treatment group in the overall study population.

At the time of the analysis, 46 patients had died. In the overall study population of nccRCC and sRCC, OS was inferior with pazopanib in comparison with sunitinib (median OS: 30.4 months with sunitinib versus 8.7 months with pazopanib, HR 2.71, 95% CI 1.31–5.58, *P* = 0.007). Three-year OS rates were 43.7% (95% CI 19.8–65.5%) with sunitinib versus 13.6% (95% CI 4.4–27.9%) with pazopanib. Kaplan–Meier curves for OS according to treatment group are presented in Figure 3.

### Subgroup analyses

Groups were unbalanced regarding histology, as shown above (Table 1). Since the poorer prognosis of unclassified and sarcomatoid histologies could influence the results, we repeated the analyses excluding these two histologies (unclassified and sarcomatoid). A total of 29 patients (papillary, chromophobe and MiT family translocation histologies) were included in the analysis.

In this subgroup analysis, treatment with sunitinib was associated with better PFS than pazopanib (HR 3.04, 95% CI 1.24–7.44, *P* = 0.015). Median PFS was 8.9 months with sunitinib and 5.1 months with pazopanib.

**Figure 4.**
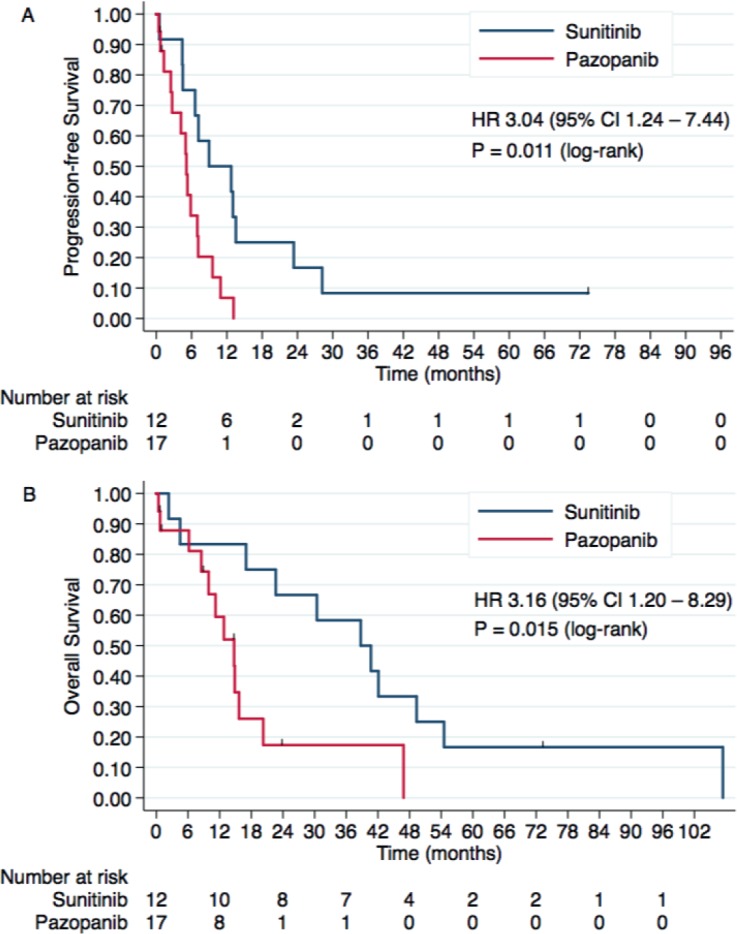
Kaplan–Meier curves for (A) PFS and (B) OS according to the treatment group in the population of nccRCC patients with papillary, chromophobe and MiT family translocation histologies.

OS was also superior with sunitinib in comparison with pazopanib (HR 3.16, 95% CI 1.20–8.29, *P* = 0.019), with a median OS of 38.7 months with sunitinib and 14.7 months with pazopanib. Three-year OS rates were 58.3% (95% CI 27–80%) and 17.3% (95% CI 2.8–42.1%), respectively. Kaplan–Meier curves for PFS and OS of this subgroup analysis (papillary, chromophobe and MiT family translocation histologies) are presented in Figure 4A and B.

In an exploratory comparison, no difference in OS according to treatment group was seen in unclassified (*P* = 0.840) and sarcomatoid histologies (*P* = 0.701). Median OS was 6.9 months in the unclassified histology group and 1.1 months in the sarcomatoid histology group.

### Factors associated with overall survival

In the overall population, factors associated with inferior OS in the univariable analysis were unclassified histologic subtype (HR 2.68, 95% CI 1.26–5.72, *P* = 0.010), sarcomatoid histologic subtype (HR 6.15, 95% CI 2.29–16.40, *P* < 0.001), poor IMDC risk (HR 5.50, 95% CI 1.26–23.94, *P* = 0.023) and treatment with pazopanib (HR 2.71, 95% CI 1.31–5.58, *P* = 0.007).

The IMDC score and TKI treatment were included in the multivariable analysis. The histologic subtype was not included because it was associated with TKI treatment (*P* = 0.023). In the multivariable analysis, poor IMDC (HR 5.77, 95% CI 1.32–25.11, *P* = 0.019) and treatment with pazopanib (HR 2.62, 95% CI 1.29–5.33, *P* = 0.008) were associated with inferior OS. Table 2 summarises the results of factors associated with OS in the overall population.

In the population excluding sarcomatoid and unclassified histologies, the presence of central nervous system (CNS) metastasis (HR 8.58, 95% CI 1.97–37.3, *P* = 0.004) and treatment with pazopanib (HR 3.16, 95% CI 1.20–8.29, *P* = 0.019) were associated with shorter OS. Both factors were also associated with inferior OS in the multivariable analysis (CNS: HR 6.56, 95% CI 1.51–28.42, *P* = 0.012; treatment with pazopanib: HR 2.87, 95% CI 1.06–7.76, *P* = 0.037). Table 3 presents the univariable and multivariable analyses of factors associated with OS in this population.

## Discussion

The present study suggests that patients with metastatic nccRCC have inferior OS when treated with pazopanib in comparison with sunitinib (median OS: 30.4 months versus 8.7 months; *P* = 0.005).

Since histologic subtypes were unbalanced between arms and unclassified and sarcomatoid histologies have poorer outcomes [20, 21], we repeated the analyses excluding these two histologies. This subgroup analysis showed that pazopanib was associated with inferior PFS (median: 8.9 months versus 5.1 months; *P* = 0.011) and OS (median: 38.7 months versus 14.7 months; *P* = 0.015) in patients with papillary, chromophobe and MiT family translocation subtypes. Multivariable analyses also supported the inferior outcomes with pazopanib. Unclassified and sarcomatoid histologies, otherwise, were associated with shorter survival, regardless of the TKI used.

For metastatic clear cell RCC, first-line therapy with pazopanib has shown to be non-inferior to sunitinib (17). Due to the extrapolation of this data to nccRCC, pazopanib has also been frequently used for these patients. As previously mentioned, sunitinib is the TKI agent with the most robust evidence for nccRCC [9, 13–16]. Prospective studies evaluating the efficacy of pazopanib are limited.

A previous retrospective cohort evaluated 37 patients with metastatic nccRCC treated with first-line pazopanib [22]. In this study, the median OS was 17.3 months, very similar to patients who received pazopanib in our study. On the other hand, a phase II single-arm trial with 29 patients with nccRCC treated with pazopanib showed more favourable outcomes, with median OS not reached and 1-year OS of 69% [23]. However, the majority of patients included had favourable Memorial Sloan-Kettering Cancer Center (MSKCC) risk, which may justify these better outcomes.

Data evaluating sRCC are scarce; however, some studies suggest poor outcomes with treatment with anti-VEGF TKIs [21, 24]. Phase III studies evaluating first-line immune-checkpoint inhibitors for metastatic RCC showed that the subgroup of patients with sRCC has high PD-L1 expression (47%–63%) [25, 26]. In the IMmotion151 trial, a study that compared the combination of atezolizumab and bevacizumab versus sunitinib, patients with sRCC had a benefit in PFS with the combination arm (HR 0.56, 95% CI 0.38–0.83) [27]. Moreover, the CheckMate214 trial showed that sRCC had encouraging response rate (57%) with nivolumab plus ipilimumab [28]. These results suggest that immune-checkpoint inhibitors might be a better therapeutic strategy for this subgroup of patients. Recently, two phase II trials have also shown some activity of immune checkpoint inhibitors for nccRCC. One evaluated atezolizumab plus bevacizumab and showed a response rate of 25% for nccRCC [29]. In the other, pembrolizumab was associated with response rates of 25.4% with papillary, 9.5% with chromophobe and 34.6% with unclassified nccRCC [30].

Certainly, in order to improve the outcomes in these subgroups of patients, the distinct behaviour of each histologic subtype needs to be recognised. To study each subtype separately, multicentre efforts are required. Hopefully, advances in the comprehension of molecular features of these rare histologies and their microenvironment will allow to better understand these different diseases and develop personalised treatments [31].

Our study has several limitations that should be considered when interpreting the results. First, this is a single-centre retrospective analysis with a small sample size. While several provocative associations have been demonstrated, causal relationships are difficult to assess. Possible bias should be considered while interpreting our results. The two treatment groups, for example, were unbalanced in terms of histologic subtype, which was an important prognostic factor. We considered this possibility and repeated the analyses after exclusion of unclassified and sarcomatoid histologies, which confirmed the results. However, other confusion factors, including unbalance for unknown factors, might have influenced the results. The intrinsic difficulties of studying nccRCC were also faced. The population included was heterogeneous, composed by histologic subtypes with diverse disease behaviour, treatment response and prognosis. Another limitation is that subsequent treatment lines for RCC are not available in the Brazilian public health system and most patients received no further treatment after progression on the first-line TKI.

On the other hand, the present study is valuable for providing new data on rare and difficult to study diseases. The new hypothesis generation is a major contribution. The large OS difference observed in our study, together with the previous literature available on sunitinib, suggests that this drug is the TKI inhibitor with the best available evidence for effectiveness in the treatment of nccRCC so far.

## Conclusions

In conclusion, in this single-centre retrospective cohort, pazopanib was associated with inferior OS in comparison with sunitinib as first-line treatment for metastatic nccRCC. However, the effectiveness of TKI treatment may differ importantly depending on the histologic subtype of nccRCC and sRCC. Ideally, this study’s findings should be confirmed prospectively by multicentre collaborative efforts with a larger number of patients.

## Authors’ contributions

RC Bonadio, PI Velho, GN Marta, M Nardo, MCLA Souza, DQB Muniz and C Dzik participated in the concept and design of the study. RC Bonadio, PI Velho, GN Marta, M Nardo, MCLA Souza, ROF Bezerra, RKA Bispo and SF Faraj participated in the data collection. ROF Bezerra, PI Velho, GN Marta, M Nardo, MCLA Souza, David QB Muniz, ROF Bezerra, RKA Bispo, SF Faraj, DA Bastos and C Dzik participated in the data analysis, drafted, reviewed and approved the final manuscript.

## Funding

This research did not receive any specific grant from funding agencies in the public, commercial or not-for-profit sectors.

## Ethical approval

The study was approved by the Local Research Ethics Committees.

## Authors’ disclosures of potential conflicts of interest

**Renata Colombo Bonadio**

Travel, Accomodation, Expenses: Roche.

**Pedro Isaacsson Velho**

Honoraria: Bayer

Speakers’ Bureau: AstraZeneca, Pfizer, Bristol-Myers Squibb

Research Funding: Bristol-Myers Squibb, Pfizer (Inst)

Expert Testimony: Bayer

Travel, Accomodations, Expenses: AstraZeneca, Astellas Pharma, Pfizer, Merck Serono, Merck

**Guilherme Nader Marta**

Travel, Accomodation, Expenses: Bayer Schering Pharma and Roche.

**Manoel Carlos Leonardi de Azevedo Souza **

Travel, Accommodations, Expenses: Zodiac Pharma

Speakers’ Bureau: MSD, BMS, Amgen.

**David QB Muniz**

Speakers’ Bureau: Janssen and Pfizer.

Research funding: Pfizer

Travel, Accomodation, Expenses: Janssen.

**Diogo Assed Bastos**

Honoraria: Janssen, Bayer, Astellas, MSD, and BMS.

Research funding: Astellas, Janssen and Pfizer.

**Mirella Nardo, Regis OF Bezerra, Raisa KA Bispo, Sheila F Faraj** and **Carlos Dzik** have no relationship to disclose.

## Figures and Tables

**Table 1.** Patients’ characteristics.

Clinical and demographic characteristics	Sunitinib *N*(%) (*N*= 16)	Pazopanib *N*(%) (*N*= 37)	Overall *N*(%) (*N*= 53)	*P*
Gender Male Female	6 (37.5) 10 (62.5)	20 (54.0) 17 (45.9)	27 (50.9) 26 (49.0)	0.372^1^
Age: median (range)	57.9 (29.3–79.1)	55.2 (16.9–77.1)	56.3 (16.9–79.1)	0.343^2^
KPS >90% 80% < 70%	9 (56.2) 4 (25) 3 (18.7)	16 (43.2) 5 (13.5) 16 (43.2)	25 (47.1) 9 (16.9) 19 (35.8)	0.193^1^
Prior nephrectomy: Yes No	9 (56.2) 7 (43.7)	18 (48.6) 19 (51.3)	27 (50.9) 26 (49.0)	0.766^1^
IMDC risk group Favourable Intermediate Poor	1 (6.2) 8 (50) 7 (43.7)	3 (8.1) 18 (48.6) 16 (43.2)	4 (7.5) 26 (49.6) 23 (43.4)	1.000^1^
No. metastatic sites: 1 2 ≥ 3	4 (25) 6 (37.5) 6 (37.5)	8 (21.6) 5 (13.5) 24 (64.8)	12 (22.6) 11 (20.7) 30 (56.6)	0.086^1^
Sites of metastatic disease Lung Lymph nodes Bone Liver CNS	10 (62.5) 12 (75) 7 (43.7) 1 (6.2) 1 (6.2)	22 (59.4) 31 (83.7) 15 (40.5) 14 (37.8) 3 (8.1)	32 (60.3) 43 (81.1) 22 (41.5) 15 (28.3) 4 (7.5)	-
Histology Papillary Chromophobe Sarcomatoid MiT family Translocation Unclassified	8 (50) 4 (25) 2 (12.5) 0 (0) 2 (12.5)	12 (32.4) 1 (2.7) 5 (13.5) 4 (10.8) 15 (40.5)	20 (37.7) 5 (9.4) 7 (13.5) 4 (10.8) 17 (32.0)	0.023^1^

KPS, Karnosky performance status; IMDC, International Metastatic Renal-Cell Carcinoma Database Consortium; No, number; CNS, central nervous system.

1 Fisher’s exact test;

2 Student *t*-test.

**Table 2.** Univariable and multivariable analyses of factors associated with OS in the overall study population.

	Univariable analysis		Multivariable analysis	
	HR (95% CI)^1^	*P*value^1^	HR (95% CI)^1^	*P*value^1^
Age (≥ 60y versus < 60)	1.05 (0.55–2.02)	0.862		
Histology Papillary Chromophobe MiT family translocation Sarcomatoid Unclassified	(reference) 0.37 (0.10–1.31) 1.68 (0.47–6.03) 6.15 (2.29–16.49) 2.68 (1.26–5.72)	0.125 0.420 <0.001 0.010		
IMDC group Favourable risk Intermediate risk Poor risk	(reference) 2.60 (0.60–11.17) 5.50 (1.26–23.94)	0.197 0.023	(reference) 2.96 (0.68–12.76) 5.77 (1.32–25.11)	0.144 0.019
No. metastatic sites 1 2 ≥3	(reference) 1.27 (0.51–3.15) 1.75 (0.80–3.83)	0.599 0.156		
CNS metastasis (yes versus no)	2.54 (0.75–8.75)	0.131		
Liver metastasis (yes versus no)	1.15 (0.57–2.29)	0.686		
Treatment (pazopanib versus sunitinib)	2.71 (1.31–5.58)	0.007	2.62 (1.29–5.33)	0.008

HR, hazard ratio; CI, confidence interval; IMDC, International Metastatic Renal-Cell Carcinoma Database Consortium; No., number; CNS, central nervous system.

1 Cox regression.

**Table 3.** Univariable and multivariable analyses of factors associated with OS in the population of patients with papillary, chromophobe and MiT family translocation histologies.

	Univariable analysis		Multivariable analysis	
	HR (95% CI)^1^	*P* value^1^	HR (95% CI)^1^	*P* value^1^
Age (≥ 60y versus < 60)	0.76 (0.27–2.07)	0.594		
Histology Papillary Chromophobe MiT family translocation	(reference) 0.35 (0.10–1.26) 1.60 (0.44–5.75)	0.112 0.467		
IMDC group Favourable risk Intermediate risk Poor risk	(reference) 2.02 (0.44–9.20) 3.49 (0.73–16.63)	0.359 0.115		
No. metastatic sites 1 2 ≥3	(reference) 0.78 (0.22–2.75) 1.80 (0.67–4.82)	0.711 0.239		
CNS metastasis (yes versus no)	8.58 (1.97–37.33)	0.004	6.56 (1.51–28.42)	0.012
Liver metastasis (yes versus no)	1.63 (0.51–5.21)	0.426		
Treatment (pazopanib versus sunitinib)	3.16 (1.20–8.29)	0.019	2.87 (1.06–7.76)	0.037

HR, hazard ratio; CI, confidence interval; IMDC, International Metastatic Renal-Cell Carcinoma Database Consortium; No., number; CNS, central nervous system.

1 Cox regression.
